# Construction of 1D conductive Ni-MOF nanorods with fast Li^+^ kinetic diffusion and stable high-rate capacities as an anode for lithium ion batteries†

**DOI:** 10.1039/c9na00616h

**Published:** 2019-11-11

**Authors:** Lingzhi Guo, Jinfeng Sun, Xuan Sun, Jinyang Zhang, Linrui Hou, Changzhou Yuan

**Affiliations:** School of Materials Science & Engineering, University of Jinan Jinan 250022 P. R. China mse_houlr@ujn.edu.cn mse_yauncz@ujn.edu.cn ayuancz@163.com

## Abstract

1D Ni-MOFs with a hexagonal honeycomb structure, good electronic conductivity and fast Li^+^ kinetic diffusion were hydrothermally prepared, and they exhibited excellent lithium storage performance in terms of high-rate reversible capacity and long-duration cycling behavior as the anode material in lithium ion batteries.

## Abstract 2

1D conductive Ni-CAT nanorods with a superb Li^+^ diffusion coefficient and electronic conductivity exhibited remarkable electrochemical lithium storage properties, and the charge-storage mechanism involved was rationally put forward.

## Graphical abstract

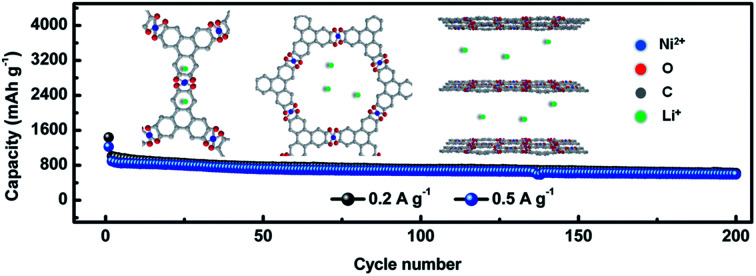

With rapid social and economic development, the ever-increasing demand for sustainable energy is apparent. Thus, extensive investigations into efficient energy storage systems for promoting the utilization of clean and renewable energy have been greatly stimulated as the research focus worldwide. Lithium ion batteries (LIBs), as the most promising energy storage devices, have more and more requirements (such as high capacity, long life, safety and environmental friendliness) for advanced electrode materials.^1^ In particular, one can note that the solid diffusion coefficient of lithium ions has become a necessary parameter for high-performance electrodes, since the Li^+^ diffusion coefficient (*D*_Li_) always influences their rate capability and the high-power-output ability of devices.^2^ The purposeful exploration of electroactive materials with fast lithium diffusion properties and satisfactory capacities is therefore of particular significance for advanced LIBs.^3^

Recently, conductive metal organic frameworks (MOFs) have drawn enormous attention as potential electrode materials because of their simple synthesis process, adjustable structure, environmental amity, and especially superb electronic conductivity.^4^ Typically, metal-catecholates (M-CATs), a class of conductive MOFs, are composed of central metal ions and 2,3,6,7,10,11-hexahydroxytriphenylene (HHTP) ligands,^5^ where metal ions are coordinated with adjacent HHTP linkers to form an extended two-dimensional (2D) framework. Meanwhile, the oxygen atoms in HHTP can also combine with axial water ligands to form hydrogen bonds.^5^ Thus, M–CAT exhibits two types of stacking structures; one is the 2D extended framework with hexagonal pores (∼1.2 nm) and a honeycomb structure *via* oxygen bonds and π–π interactions, where metal nodes and organic linkers serving as charge carriers enable full charge delocalization in the 2D plane, resulting in good electrical conductivity,^6^ and the other is the stacking along the *c*-axis *via* hydrogen bonds, which forms a connection between the layers, easily forming one-dimensional (1D) structures.^5^ Owing to its unique porous structure and good electrical conductivity, the M–CAT holds enormous promise in wide energy-related applications, such as catalysis,^7^ supercapacitors^8^ and LIBs.^9^ However, the intrinsic charge-storage mechanisms of the M–CAT electrodes have rarely been studied so far.

In this work, we reported the hydrothermal synthesis (more details described in the Experimental section, see the ESI†) of 1D conductive Ni-CAT nanorods (NRs) towards LIB applications. As a potential anode for LIBs, the involved lithium storage mechanism of the Ni-CAT NRs was tentatively put forward here. Furthermore, the as-synthesized Ni-CAT NRs exhibited remarkable *D*_Li_ values in the order of 10^−9^ to 10^−10^ cm^2^ s^−1^ during the intercalation/de-intercalation process and delivered a large discharge capacity of ∼889 mA h g^−1^ at 0.1 A g^−1^ as well as good rate capability with ∼428 mA h g^−1^ at 2.0 A g^−1^ and remarkable cycling stability even at high rates.

Fig. 1a shows the field emission scanning electron microscopy (FESEM) image of the obtained Ni-CAT sample. As visualized, Ni-CAT exhibits a uniform 1D NR architecture of ∼500 nm in length and ∼50 nm in diameter, which can be well supported by the transmission electron microscopy (TEM) observation (Fig. 1b). The scanning TEM (STEM) image and the corresponding energy dispersive X-ray spectroscopy (EDS) mapping images (Fig. 1c–f) evidence the even distribution of elemental Ni, O and C throughout the NRs.

**Fig. 1:**
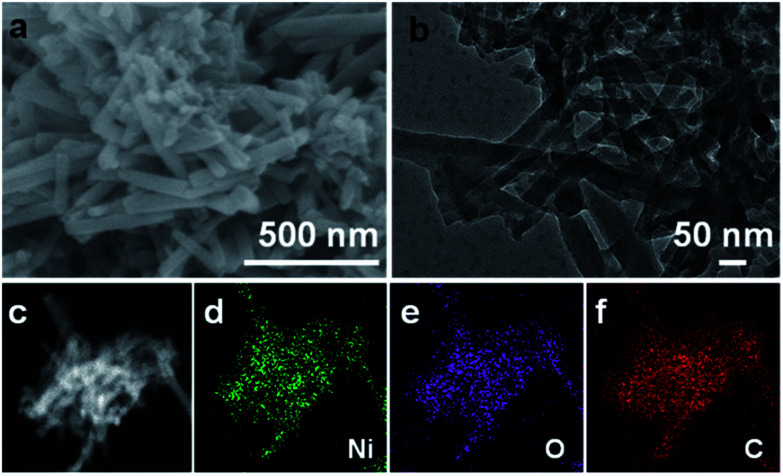
(a) FESEM, (b) TEM, (c) STEM and corresponding EDS elemental (d) Ni, (e) O and (f) C mapping images of the Ni-CAT NRs.

The typical crystal structure of Ni-CAT, as shown in Fig. 2a, demonstrates that each Ni(ii) is combined with two HHTP molecules to form a hexagonal honeycomb structure. The porosity of Ni-CAT NRs is evaluated by Brunner–Emmett–Teller (BET) measurements (Fig. S1a and b, ESI†). A specific surface area of ∼85.8 m^2^ g^−1^ and pore volume of ∼0.43 m^3^ g^−1^ can be detected for the NRs. The co-existence of micropores and mesopores (Fig. S1b, ESI†) would provide more active sites and buffer the volume changes during cycling.^10^ X-ray diffraction (XRD) analysis reveals the good crystalline character of the Ni-CAT NRs, as depicted in Fig. 2b. The peaks located at 2*θ* = ∼4.7, ∼9.5, ∼12.6 and ∼16.5° illustrate the long-range order of the crystal in the *ab* plane, and the distinct reflection at ∼27.3°, corresponding to the (001) plane, implies the short-range order of the *c* axis, indicating that the resultant Ni-CAT is a covalently linked layered material.^11^

**Fig. 2:**
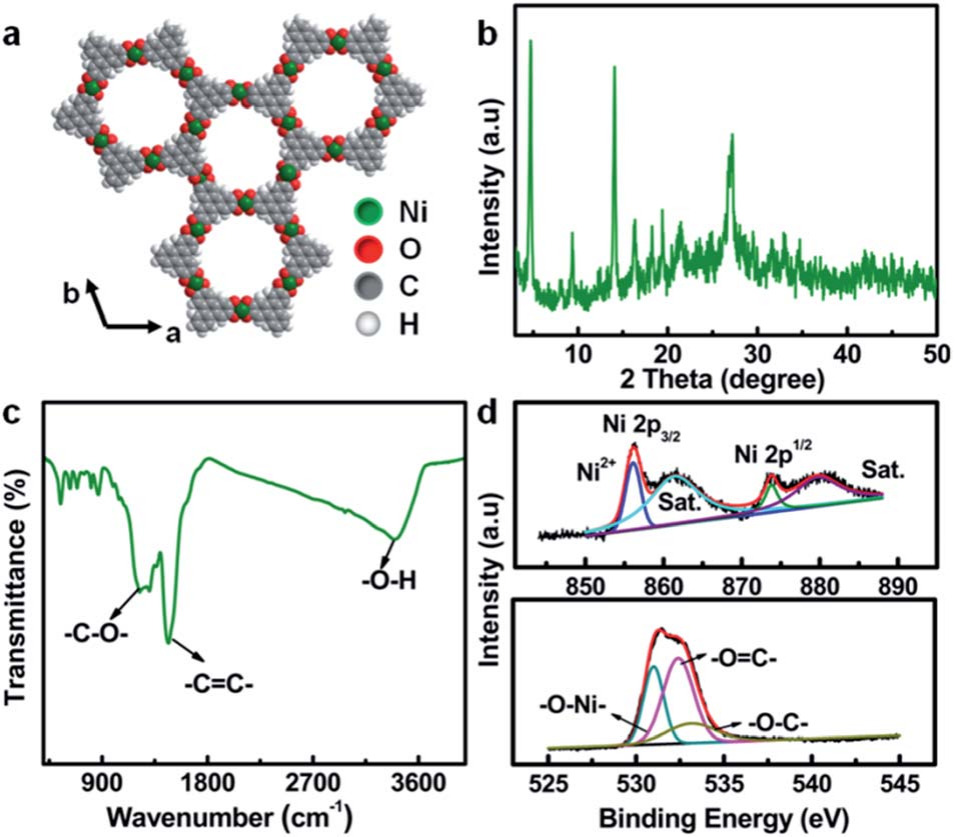
(a) Crystal structure of Ni-CAT; (b) XRD pattern, (c) FT-IR spectrum, and (d) XPS Ni 2p (top) and O 1s (bottom) spectra of the Ni-CAT NRs.

In order to further analyze the phase structure of Ni-CAT, Fourier transform infrared spectroscopy (FT-IR) was conducted, as presented in Fig. 2c. Typically, the bonds at 1219, 1474 and 3373 cm^−1^ are attributed to the –C=C– stretching, –C–O– stretching vibration, and –O–H, respectively,^12,13^ fully proving the existence of multiple organic functional groups related to organic HHTP. Meanwhile, the X-ray photoelectron spectroscopy (XPS) full-scan spectrum (Fig. S2, ESI†) shows the existence of Ni, C and O elements in the synthesized Ni-CAT NRs. Fig. 2d exhibits the high-resolution XPS spectra of Ni 2p (top) and O 1s (bottom). The peaks at 856.1 and 873.8 eV are ascribed to Ni 2p_3/2_ and 2p_1/2_, respectively, along with two satellite (Sat.) peaks at 861.4 and 879.7 eV, indicating the presence of bivalent Ni in the Ni-CAT NRs.^12^ Besides, the O 1s spectrum can be deconvoluted into three peaks at 531.0, 532.4 and 533.3 eV, which represent O–Ni, O=C and O–C, respectively.^12^ The detailed physicochemical characterization and analysis above confirm the successful formation of the 1D Ni-CAT NRs.

As has been well established, the solid phase diffusion coefficient is one of the important indices for LIBs because solid phase diffusion is a voltage transient process.^2^ The galvanostatic intermittent titration technique (GITT) is an effective and credible approach to evaluate the solid phase diffusion properties.^2^Fig. 3a shows the GITT profile of the Ni-CAT anode at a current density of 0.5 A g^−1^, where the charge time (*t*) is 200 s and the rest time (*τ*) is 150 s (the details are given in Fig. S3, ESI†). Based on this, the *D*_Li_ is calculated during the discharge (Fig. S3a and b, ESI†) and charge (Fig. S3c and d, ESI†) processes. As plotted in Fig. 3b, the *D*_Li_ values of the Ni-CAT NRs are in the order of 10^−9^ cm^2^ s^−1^ for the discharge case, which is much higher than those of other anodes including hollow Fe–Mn–O/C microspheres (∼10^−12^ cm^2^ s^−1^),^10^ N-doped yolk–shell carbon nanocages filled with ZnSe/CoSe_2_ nanodots (∼10^−15^ cm^2^ s^−1^),^14^ hollow NiO@Co_3_O_4_@graphene quantum dot spheres (∼10^−15^ cm^−2^ s^−1^),^15^ ZnCo_2_O_4_@carbon nanotubes (∼10^−11^ cm^−2^ s^−1^),^16^ and so on. Notably, the changing trend of the *D*_Li_ values during charging (Fig. 3c) is similar to that for the discharge process (Fig. 3b). However, the *D*_Li_ values are in the order of 10^−10^ cm^2^ s^−1^, which indicates the slower Li^+^ de-intercalation behaviour. The fast lithium diffusion in the Ni-CAT NRs may be attributed to the 1D porous MOF structure, which provides more efficient diffusion channels for Li^+^ ions^17,18^ and will benefit the large reversible capacities especially under high current densities.^2^

**Fig. 3:**
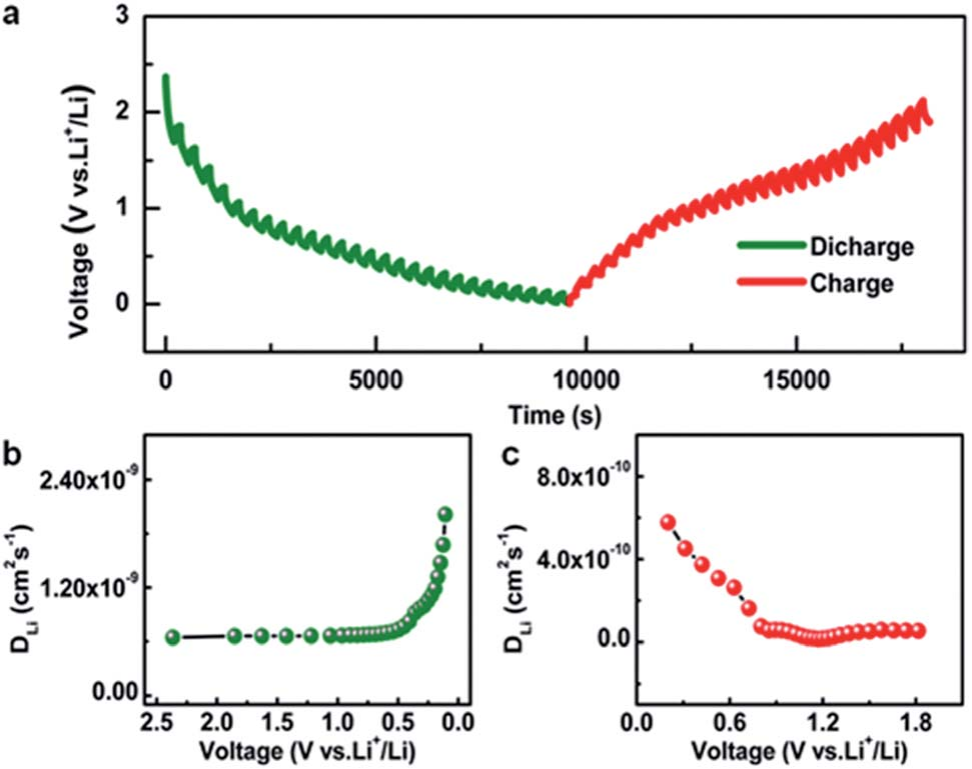
(a) GITT curves for discharge/charge processes and *D*_Li_ values at various (b) discharge and (c) charge states of the Ni-CAT NR electrode.

The electrochemical properties of Ni-CAT are elaborately shown in Fig. 4. The cyclic voltammetry (CV) curves of the Ni-CAT NRs were obtained from 0.01 to 3.0 V (*vs.* Li^+^/Li) at a scanning rate of 0.1 mV s^−1^ (Fig. 4a). In the first negative sweep, a large broad peak appears at 1.24 V and then vanishes in the following scans, which is probably caused by the formation of a solid electrolyte interphase (SEI) film. The weak peak at 0.58 V can be attributed to the partial decomposition of carbonate electrolytes.^19^ In the second cathodic sweep, there are a series of small humps at 0.62–1.49 V due to the Li^+^ insertion process. And the peaks located between 0.98 and 1.32 V in the anodic scan correspond to the de-insertion process. Furthermore, the well-overlapped CV curves of the 2^nd^ and 3^rd^ cycles corroborate the striking electrochemical reversibility of the Li^+^-insertion/de-insertion processes. It is worth mentioning that the binding energies of Ni 2p in the cycled Ni-CAT NRs are not changed (Fig. S4a, ESI†), indicating the good preservation of the central Ni^2+^ ions during cycling, which further affirms that the Ni species is not involved in the charge storage process of the Ni-CAT at all. Meanwhile, compared with the C 1s spectrum of the Ni-CAT NRs before and after 50 cycles (Fig. S4b†), it is easy to find a weak peak appearing at 289.8 eV, which corresponds to the C–Li groups and can be ascribed to the lithiation reaction with C=C groups.^20^ Therefore, lithium storage in Ni-CAT mainly relies on the reversible insertion/de-insertion in its organic ligands and pores (Fig. S5, ESI†). Furthermore, with more and more lithium ions inserting into the benzene rings (Fig. S5, ESI†), the repulsion between the charges will also increase, leading to the weak peaks observed in the CV curves (Fig. 4a), similar to the previous report.^14^ Meanwhile, lithium ions may also insert into the interlaminar spacing (∼0.37 nm) in the *c*-axis direction,^21^ as confirmed by the *ex situ* XRD data (Fig. S6, ESI†). The XRD peaks of the Ni-CAT electrode change significantly during the discharge process. The main peaks are weakened and even disappear with discharging. In contast, after Li^+^ de-insertion, the structure of Ni-CAT NRs gradually recovers, but the reflection at 2*θ* = ∼27.3° still cannot be observed, which indicates the destruction of short-range order in the *c*-axis direction owing to Li^+^ insertion. Thus, there are three potential lithium storage sites, including the (I) benzene rings, (II) pores and (III) interlaminar space (Fig. 4b).

**Fig. 4:**
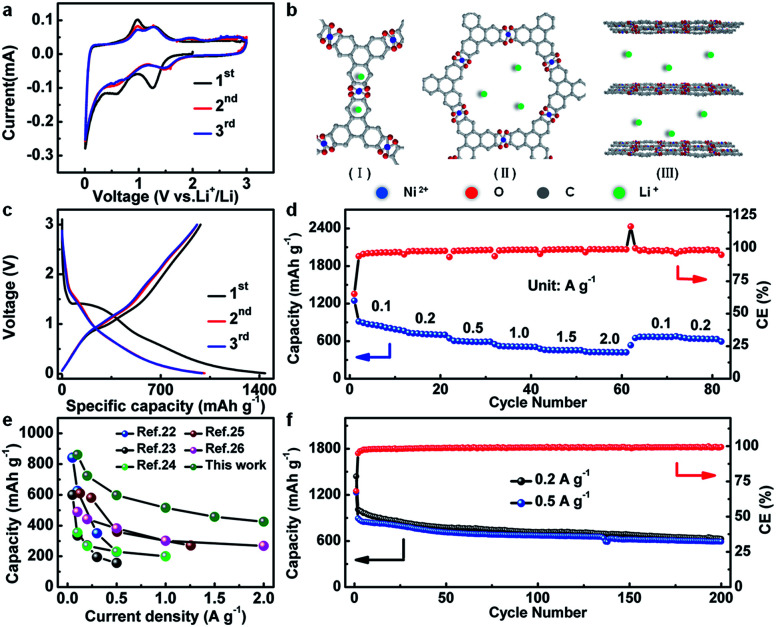
(a) CV curves (0.1 mV s^−1^), (b) potential lithium-storage sites (I, benzene rings; II, pores; III, interlaminar space), (c) initial three charge and discharge plots (0.2 A g^−1^), (d) rate behaviour, (e) comparison of capacities with other anodes at various current densities, and (f) cycling properties at 0.2 and 0.5 A g^−1^ of the Ni-CAT NRs.

Fig. 4c shows the discharge–charge plots of the Ni-CAT NRs in the potential range of 0.01–3.0 V at a current density of 0.2 A g^−1^. During the first cycle, the initial charge and discharge capacities are ∼982 and ∼1440 mA h g^−1^, respectively, corresponding to a coulombic efficiency (CE) of ∼68%. The capacity loss here can be ascribed to the formation of the SEI film and the irreversible Li extraction process.^19^ Additionally, the discharge capacities of the 2^nd^ and 3^rd^ cycles still remain as large as ∼1010 and ∼993 mA h g^−1^, respectively.

Besides, the rate performance of the Ni-CAT anode is shown in Fig. 4d. More impressively, the average discharge capacities are estimated to be ∼889, ∼654, ∼610, ∼526, ∼464 and ∼428 mA h g^−1^ at different current densities of 0.1, 0.2, 0.5, 1.0, 1.5 and 2.0 A g^−1^, respectively. When the current decreases in turn to 0.1 and 0.2 A g^−1^, the reversible capacities can be rapidly recovered to ∼652 and ∼648 mA h g^−1^, respectively. As depicted in Fig. 4e, the reversible capacities of our Ni-CAT are even larger than those of other previously reported MOF-based anodes, particularly at high current densities, for example, terephthalic acid based Ni-MOF (∼229 mA h g^−1^ at 2.0 A g^−1^),^22^ Co-isophthalic MOF (∼157 mA h g^−1^ at 0.5 A g^−1^),^23^ Pb(4,4′-ocppy)_2_ (∼268 mA h g^−1^ at 2.0 A g^−1^),^24^ Ni/Mn-1,3,5-benzenetricarboxylic acid (∼270 mA h g^−1^ at 1.5 A g^−1^),^25^ Cd(1,1′,1′′-(1,3,5-triazine-2,4,6-triyl)tripiperidine-4-carboxylic acid) (∼200 mA h g^−1^ at 1.0 A g^−1^),^26^ Co_3_(HHTP)_2_ (∼380 mA h g^−1^ at 2.0 A g^−1^),^27^ and Cu-CAT (∼381 mA h g^−1^ at 2.0 A g^−1^).^28^ This convincingly highlights the remarkable advantages of 1D porous Ni-CAT NRs for high-power LIBs, thanks to their intrinsically superb Li diffusion and electronic conductivity.^29,30^

Fig. 4f shows the cycling performance of the Ni-CAT electrode at current densities of 0.2 and 0.5 A g^−1^. Appealingly, the Ni-CAT NRs maintain a reversible capacity of ∼626 mA h g^−1^ after 200 cycles at 0.2 A g^−1^. More strikingly, the NR electrode can even maintain a capacity as large as ∼592 mA h g^−1^ after the same number of cycles at a high current rate of 0.5 A g^−1^. Moreover, the CE values for each cycle approach 100% except for the first cycle. Meanwhile, the structure of Ni-CAT NRs is relatively stable even after 300 cycles. The TEM observations (Fig. S7, ESI†) show that the *ab* plane remains stable, and that only the *c* axis is somewhat damaged, which makes the length of Ni-CAT NRs further shortened, in good agreement with the *ex situ* XRD analysis above (Fig. S6, ESI†). Apparently, the 1D Ni-CAT NRs are indeed a potential anode material for high-power LIBs.

In summary, the investigation here mainly reports the hydrothermal synthesis of 1D porous conductive Ni-CAT NRs as anode materials for high-performance LIBs. More significantly, the underlying Li^+^-storage mechanism of Ni-CAT was rationally proposed. The electrically conductive Ni-CAT NRs were appealingly endowed with a high Li^+^ diffusion coefficient. As a result, the as-synthesized Ni-CAT NRs exhibited large reversible capacities of ∼889 mA h g^−1^ at 0.1 A g^−1^ and even ∼428 mA h g^−1^ at a high rate of 2.0 A g^−1^, and exceptional long-term cycling behaviour with a capacity of ∼592 mA h g^−1^ maintained over 200 consecutive cycles at 0.5 A g^−1^. The work will inspire future versatile design and construction of novel conductive MOFs for energy-related applications.

## Conflicts of interest

There are no conflicts to declare.

## Supplementary Material

NA-001-C9NA00616H-s001
