# Supplementary material for: Construction of 1D conductive Ni-MOF nanorods with fast Li+ kinetic diffusion and stable high-rate capacities as an anode for lithium ion batteries
Source: Nanoscale Adv. 2019 Nov 11;1(12):4688–91. doi: 10.1039/c9na00616h (PMC9416851; doi:10.1039/c9na00616h)
Supplement: NA-001-C9NA00616H-s001 [file NA-001-C9NA00616H-s001.pdf]

Electronic Supplementary Material (ESI) for Nanoscale Advances.
This journal is © The Royal Society of Chemistry 2019

Electronic supplementary information for:

Construction of 1D conductive Ni-MOF nanorods with fast Li^+ kinetic diffusion and stable high-rate capacities as an anode for lithium ion batteries

Lingzhi Guo,‡ Jinfeng Sun‡, Xuan Sun, Jinyang Zhang, Linrui Hou,* and Changzhou Yuan *

School of materials science & engineering, university of Jinan, Jinan, 250022, P. R. China.

E-mail: mse_houlr@ujn.edu.cn (Prof. L. Hou)

mse_yauncz@ujn.edu.cn; ayuancz@163.com (Prof. C. Yuan)

Experimental section

Materials Synthesis: Typically, 30 mg of nickel acetate (Sinopharm Chemical Reagent Co., Ltd.) monohydrate and 21 mg of 2, 3, 6, 7, 10, 11-heahydroxytriphenylene (HHTP) (95 wt.%, J&K) were added in 12 mL of a solvent mixture of de-ionized (DI) water. After ultra-sonicated for 30 min, the solution was transferred into an electric oven at 85 °C for 12 h. After washed with sufficient DI water and acetone, the Ni-CAT was obtained.

Materials Characterization: The samples was characterized by field-emission scanning electron microscopy (FESEM, JEOL-6300F), transmission electron microscopy (TEM), scanning TEM (STEM), and high-resolution TEM (HRTEM) (JEOL JEM-2100) coupled with energy dispersive X-ray spectroscopy (EDS). The X-ray diffraction (XRD) was collected by the BREKER-D8ADVANCE with a scanning rate of 20 ° min^{-1}. The specific surface areas with pore size distributions were tested on Autosorb-IQ/MP surface area analyzer (Quantachrome, America). The Fourier transformed infrared spectroscopy (FT-IR) spectrum was collected by Nicolet iS50. The X-ray photoelectron spectroscopy (XPS) was obtained by an X-ray photoelectron spectrometer (thermo ESCALAB 250XI).

The working electrode was prepared by mixing active material, carbon black, carboxymethylcellulose sodium (CMC) with a mass ratio of 7 : 2 : 1. The half cells was fabricated in a glove box filled Ar using a 2032-type coin cell with the mass loading of ~1 mg cm^{-2}. The charge-discharge tests were conducted on LAND (CT2001A) in the voltage range between 3.0 and 0.01 V. And the cyclic voltammetry (CV) was conducted on performed on an electrochemical workstation (IviumStat. h, the Netherlands).

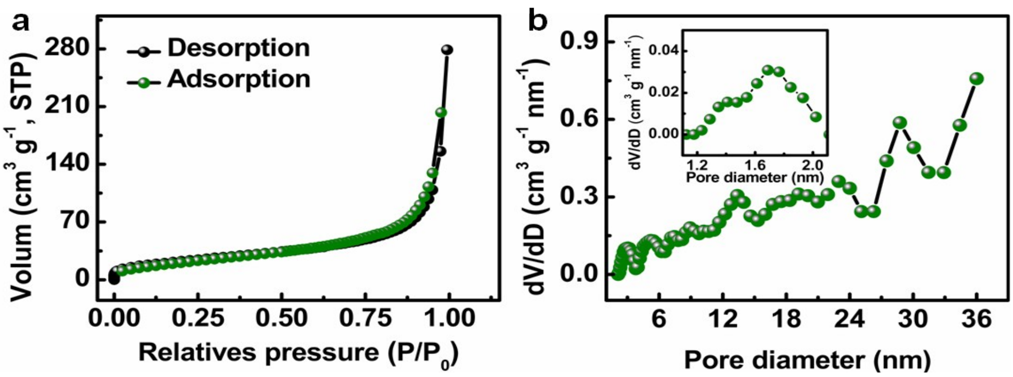

Fig. S1 (a) Nitrogen adsorption-desorption isotherms and (b) meso-pore size distribution curves of the Ni-CAT NRs. The inset for the micro-pore size distribution plots

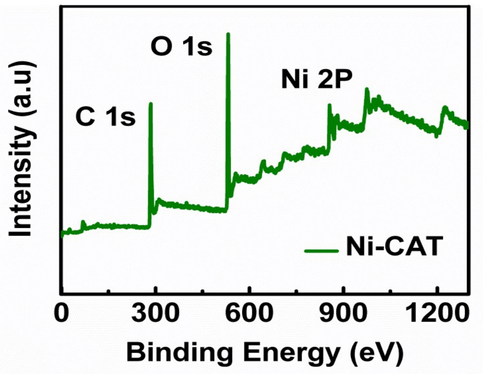

Fig. S2 XPS full scanning spectrum of the Ni-CAT NRs

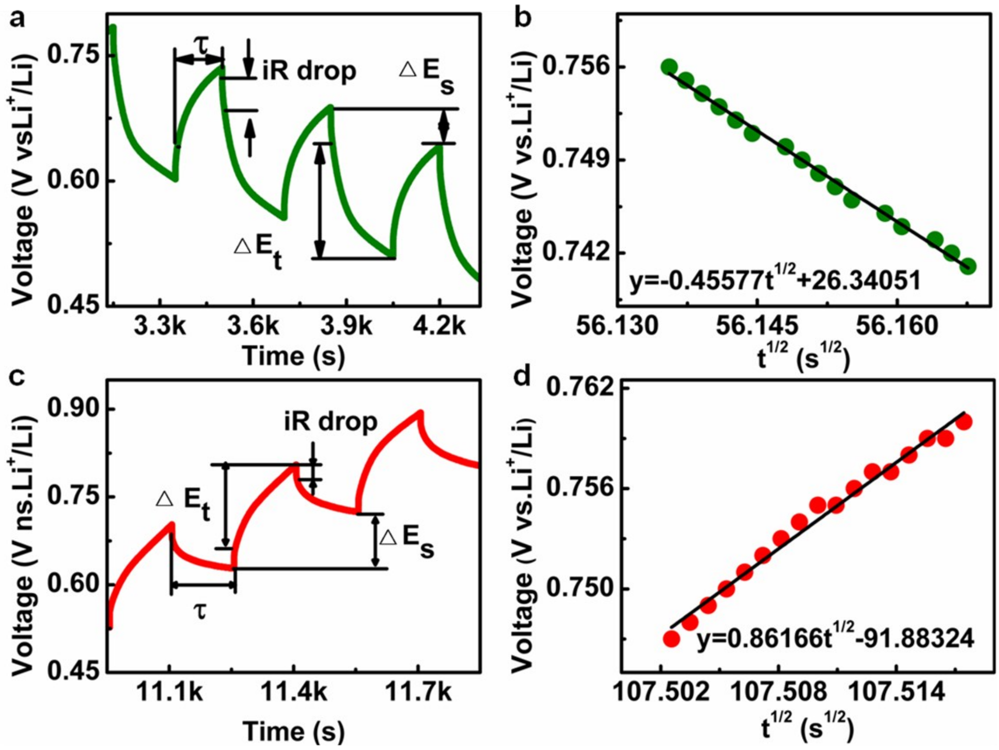

Fig. S3 (a) Detailed schematic diagram and (b) E vs. $t^{1/2}$ relationship during the discharging process; (c) Detailed schematic diagram and (d) E vs. $t^{1/2}$ relationship of during the charging process for the Ni-CAT NRs

The value of D_{Li} is calculated by the following Equation:[1]

$$D_{Li^+} = \frac{4}{\pi\tau}\left(\frac{m_B V_m}{M_B S}\right)^2\left(\frac{\Delta E_s}{\Delta E_t}\right)^2 \qquad \tau = \frac{L^2}{D_{Li^+}}$$

In the equation, the m_B is the mass of the active materials, M_B the molar mass of the electrode, V_M the molar volume, S the electrode contact area. ΔE_s the change in the change of the steady-state potential, and ΔE_t the voltage change in the discharge pulse.

The curves of E vs $t^{1/2}$ are the details of individual current pulses applied over time duration with a curve of the voltage response. This can be used as the proper value for the calculation of the D_{Li}.

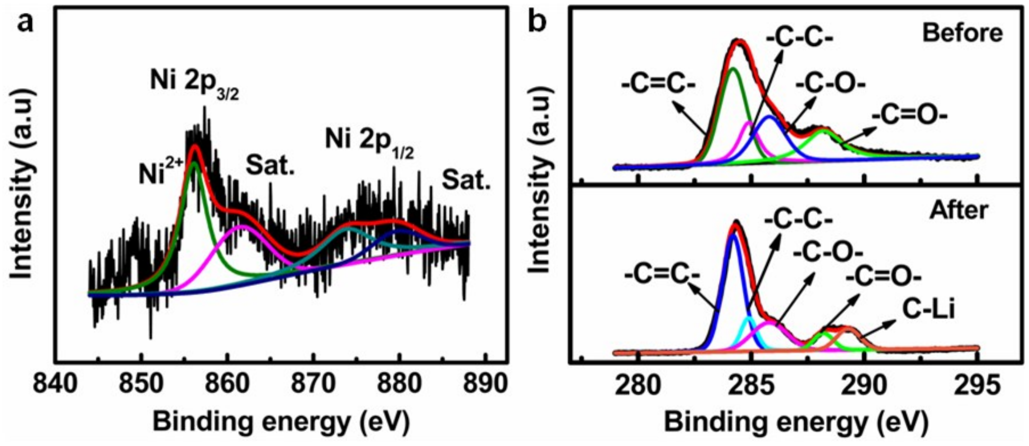

Fig. S4 (a) XPS spectra of Ni 2p after the 50th cycle; (b) XPS spectra of the C 1s before (the upper) and after (the lower) the 50th cycle for the Ni-CAT NRs

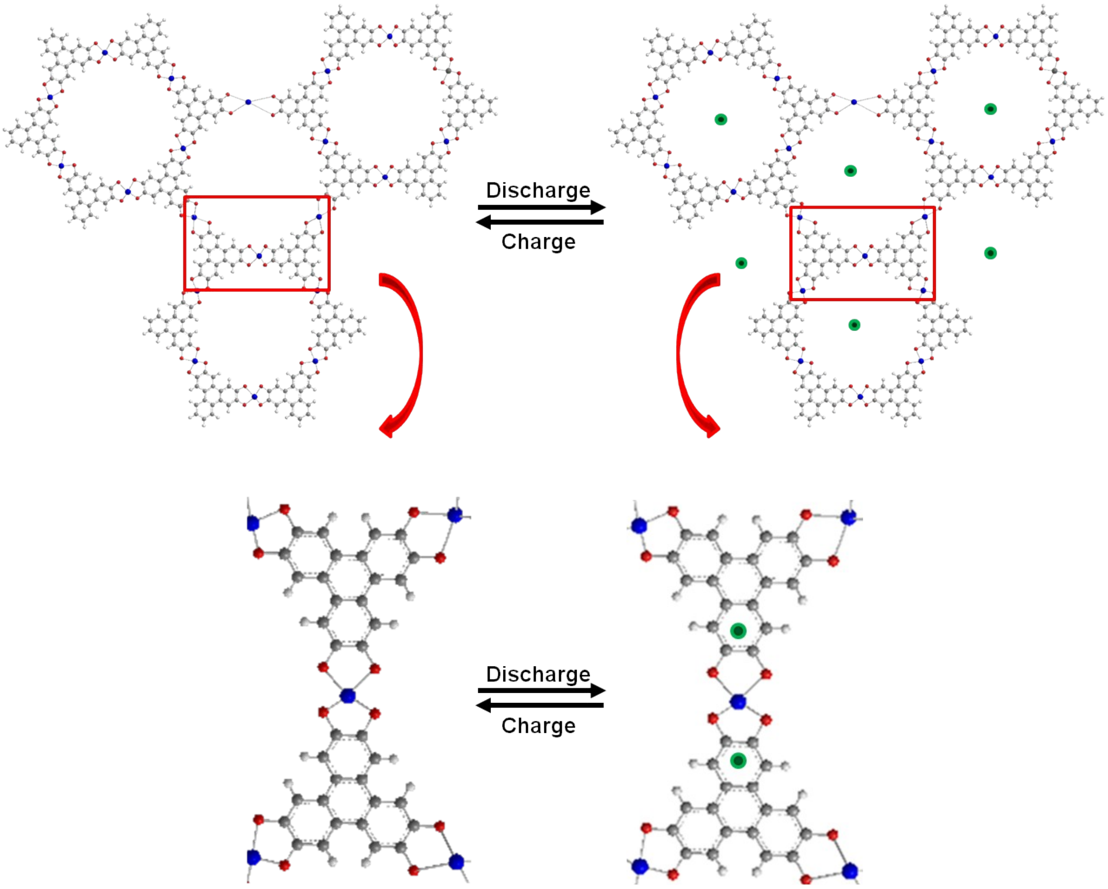

Fig. S5 Probable lithiation/de-lithiation sites for the lithium ions in the Ni-CAT. Color scheme: Ni, blue; O, red; C, gray; Li, green and H, white.

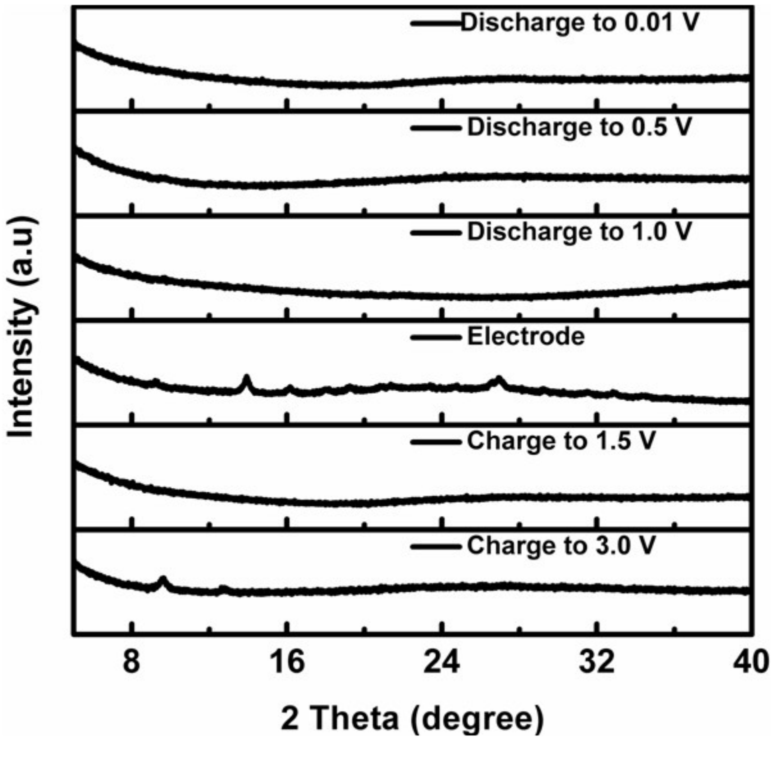

Fig. S6 Ex-situ XRD patterns collected during the first charge-discharge cycle.

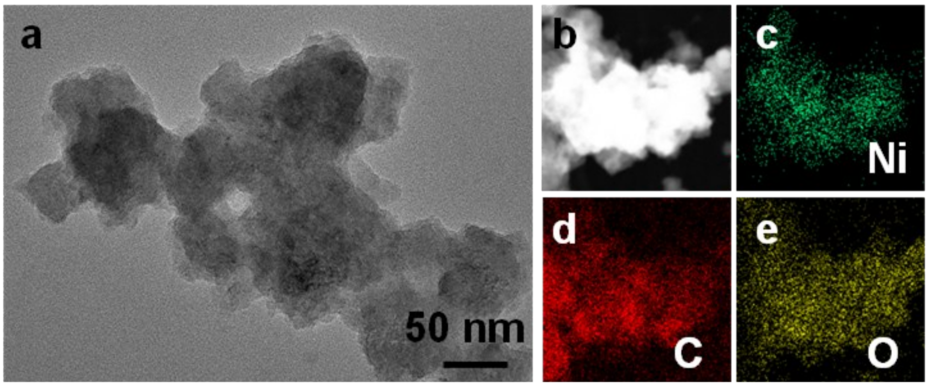

Fig. S7 (a) TEM, (b) STEM and corresponding EDS elemental (c) Ni, (d) C and (e) O mapping images of the Ni-CAT NRs after 300 cycles.

References

1 E. Allcorn, S. O. Kim and A. Manthiram, Phys. Chem. Chem. Phys, 2015, **17**, 28837.
